# Thermal Performance of Medium and Long-Wave Infrared Emitters in PEEK-Based Thermoplastic Polymer Composites

**DOI:** 10.3390/polym18050579

**Published:** 2026-02-27

**Authors:** Mehmet Emre Burulday, Nader Javani

**Affiliations:** Faculty of Mechanical Engineering, Yildiz Technical University, Istanbul 34349, Türkiye

**Keywords:** infrared heating, thermoplastic composite, PEEK-CFRTP, ceramic emitter, quartz tungsten emitter, surface temperature, thermal uniformity, energy efficiency

## Abstract

Carbon Fiber Reinforced Thermoplastic Polymer (CFRTP) composites, particularly those utilizing Polyetheretherketone (PEEK) matrices, are becoming more demanding in the automotive and aerospace industries because of their outstanding strength, resilience to impact, and capacity for recycling. The employed heating methodology to prepare these materials is important both to improve them through uniform temperature distribution and to manage the energy consumption. The current study aims to address the encountered issues by experimentally comparing the radiative–thermal performance of medium-wave (1.4–2.5 µm) Quartz Tungsten (QTM) and long-wave (3.5–5.5 µm) Ceramic (FFEH) infrared emitters using a modular laboratory-scale heating system. While QTM emitters provided rapid heating rates, they induce significant through-thickness thermal gradients and surface degradation risks due to spectral mismatch with the polymer. In contrast, long-wave Ceramic emitters demonstrate superior spectral compatibility with PEEK, expanding the safe processing window and achieving complete melting at 343 °C with high thermal uniformity and approximately 18% lower effective energy demand compared to QTM systems. Furthermore, the structural integrity of the consolidated laminates has been validated through tensile testing, yielding an average tensile strength of 873 MPa and a tensile modulus of 56.3 GPa. These findings confirm the importance of optimizing the emitter wavelength not only for energy efficiency, but also for ensuring matrix integrity and mechanical performance in high-performance composite manufacturing.

## 1. Introduction

Carbon fiber reinforced thermoplastic (CFRTP) composites represent a transformative class of advanced structural materials that are increasingly adopted in aerospace, automotive and biomedical applications due to their high specific stiffness and strength, superior damage tolerance and recyclability compared with conventional thermoset systems [1]. Among high-performance thermoplastic matrices, polyetheretherketone (PEEK) is particularly attractive because of its outstanding thermal stability (melting temperature ≈ 343 °C), tensile strengths exceeding 100 MPa and excellent chemical and environmental resistance [2]. Its semi-crystalline structure, high glass transition temperature (~141 °C) and good dimensional stability under thermal cycling make PEEK-based CFRTP laminates suitable for long-term service in harsh environments. At the same time, these properties impose strict processing windows. Insufficient heating leads to incomplete melting and consolidation, whereas excessive temperatures cause thermal degradation. As a result, reliable manufacturing of PEEK-CFRTPs requires precise thermal management and controlled heat input. Similar challenges regarding thermal history and processing conditions are observed in additive manufacturing and curing of advanced polymers, where parametric optimization is essential for mechanical performance [3,4,5]

Conventional heating techniques employed in thermoplastic composite production, such as convection ovens and contact heating plates, frequently experience extended usage time, uneven temperature distributions, and elevated energy requirements [6]. These limitations are particularly pronounced for thick or geometrically complex PEEK-CFRTP parts, where through-thickness temperature gradients can result in regions of incomplete melting, void formation, and residual stresses. Although alternative consolidation techniques such as resistance welding [7] and induction heating [8] have been studied, they often require complex tooling setups. The inherently anisotropic thermal conductivity of carbon fiber reinforcements further complicates heat transport, as heat flows preferentially along the fiber direction rather than through the laminate thickness, leading to hot spots and thermal stress concentrations that can compromise structural integrity [9]. In industrial environments, such drawbacks translate into reduced throughput, higher energy costs and challenges in guaranteeing consistent quality.

Infrared (IR) heating has therefore emerged as a promising alternative for thermoplastic composite processing, offering contactless energy delivery, high heating rates and the possibility of tailoring the spectral output of the emitters to match the absorption characteristics of the polymer matrix [10]. Controlling heating settings is necessary for the widespread use of infrared technology in a variety of industries, from industrial heating to agricultural drying [11,12]. By directly irradiating the laminate surface, IR systems can significantly shorten heating times compared with convective ovens [13]. Medium-wave quartz tungsten (QTM) emitters operating in the 1.4–2.5 μm range provide high power density and fast response, making them attractive for rapid heating applications, whereas long-wave ceramic emitters operating in the 3.5–5.5 μm band are often associated with improved temperature uniformity and more gentle heating profiles suited to high-performance polymers [14]. The overall effectiveness and energy efficiency of an IR heating configuration depend on multiple coupled factors, including emitter wavelength, power density, heater-to-surface distance, exposure duration and the optical–thermal properties of the composite. PEEK exhibits strong absorption bands in the mid-infrared region, which can be exploited by appropriate emitter selection to enhance heating efficiency [15]. Previous studies have reported energy savings of about 15–25% when optimized IR setups are used instead of conventional heating methods [16,17].

Despite these advances, several important knowledge gaps remain for PEEK-based CFRTPs. Many IR heating studies have focused on thermoset composites or lower-temperature thermoplastics, and often emphasize process feasibility rather than quantitative evaluation of thermal uniformity and energy consumption [18,19,20,21,22]. In addition, while heat transfer in thermoplastic composites has been investigated numerically—for example, Jena et al. [23] simulated temperature distributions in PEEK laminates during automated fiber placement, while other numerical studies have focused on optimizing lamp configurations and powder impregnation processes [22,24]. Limited experimental studies are available comparing different IR emitter types for PEEK-CFRTP processing under controlled conditions. In particular, there is a lack of systematic data on how emitter wavelength and spatial configuration influence temperature homogeneity, energy usage and surface melting quality in modular IR oven systems.

The present study aims to address these gaps by performing a quantitative spectral matching analysis comparing medium-wave quartz tungsten and long-wave ceramic IR emitters for the heating of PEEK-based thermoplastic polymer composites. Unlike previous studies, this work explicitly defines the trade-offs between heating rate, energy demand, and degradation risk, validated by the mechanical performance of the consolidated parts. The specific objectives of the current study are:

- To evaluate the influence of emitter type and process parameters on surface temperature evolution and PEEK melting behavior;
- To quantify and compare the energy consumption and heating efficiency based on spectral compatibility
- To assess the relationship between heating conditions and surface morphology
- To derive practical guidelines for the selection of IR emitters for energy-efficient processing. The insights obtained are expected to support the design of scalable IR-assisted manufacturing routes for high-performance thermoplastic composites.

## 2. Materials and Methods

This study aims to optimize the infrared (IR) heating process for the production of carbon fiber reinforced thermoplastic composites (CFRTPs), focusing specifically on polyetheretherketone (PEEK)-based systems. A modular infrared heating platform was designed and constructed to conduct controlled experiments with varying IR emitter types, heater-to-sample distances, and exposure durations.

### 2.1. Materials

The composite specimens were fabricated using high-performance carbon fiber reinforcement and a thermoplastic matrix specifically selected for aerospace applications. The thermoplastic matrix used in this study was Victrex^®^ 150XF-grade Polyetheretherketone (PEEK) powder (Victrex plc, Lancashire, UK), specifically designed for coating and impregnation applications with a low melt viscosity to facilitate fiber wetting. Prior to matrix application, the semi-crystalline PEEK powder was dried in a laboratory oven at 120 °C for 4 h to eliminate absorbed moisture and minimize void formation during the melting process.

The reinforcement was DowAksa DW 284 Sateen 5HS carbon fiber fabric (DowAksa, Yalova, Türkiye), a 5-harness satin weave providing high conformability and mechanical performance. The fabric specimens were cut to average dimensions of 100 × 100 mm^2^ for all experiments. The semi-pregs were produced by powder scattering followed by infrared heating.

### 2.2. Material Characterisation

To ensure the suitability of the raw materials for infrared processing, thermal and spectral characterizations were performed prior to composite fabrication. Material characterization was performed to confirm the thermal and chemical properties of the PEEK matrix and its compatibility with IR heating.

**Figure 1:**
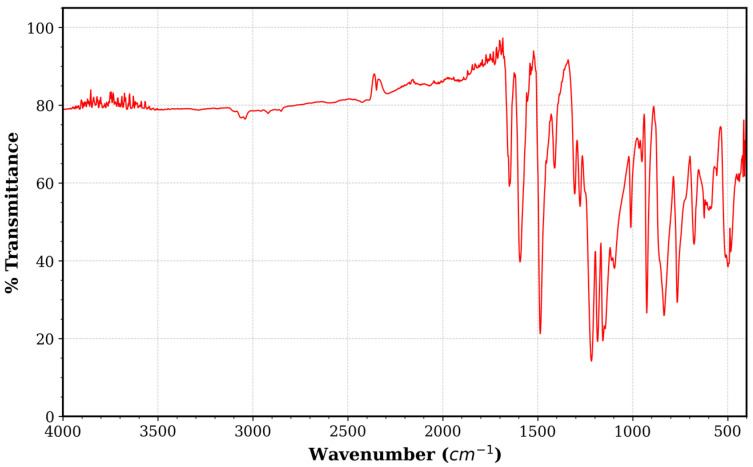
FTIR transmittance spectrum of the semi-crystalline PEEK powder used in this study.

FTIR Analysis: Fourier-transform infrared spectroscopy (FTIR) was performed using a Thermo Scientific Nicolet iS50 FTIR spectrometer (Thermo Fisher Scientific, Waltham, MA, USA) to analyze the absorption characteristics of the PEEK powder and verify its compatibility with the tested IR emitters. The spectra were collected in the range of 4000–400 cm^−1^. As shown in Figure 1, the spectrum reveals high transparency in the Near-IR region (4000–3000 cm^−1^) and significant absorption bands in the Mid-to-Far IR region (<1700 cm^−1^), indicating distinct spectral selectivity relevant to IR emitter types.

**Figure 2:**
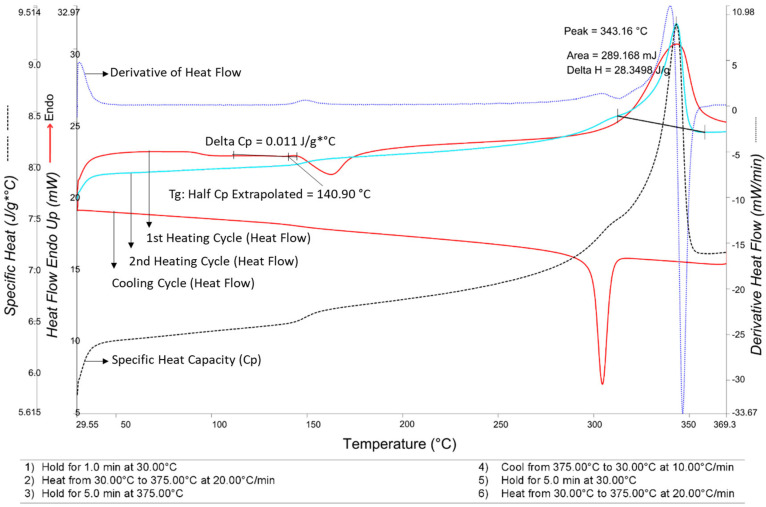
Differential Scanning Calorimetry (DSC) thermogram of the PEEK powder. The plot displays multiple thermomagnetic properties: the top and bottom red solid curves represent the first heating and cooling cycles, respectively. The turquoise solid curve represents the second heating cycle used to eliminate thermal history. The blue dashed line indicates the derivative of the heat flow, and the black dashed line shows the specific heat capacity. The precise calculation of the glass transition temperature and the change in specific heat are explicitly illustrated on the first heating curve.

DSC Analysis: Differential Scanning Calorimetry (DSC) analysis was conducted using a PerkinElmer DSC 4000 (PerkinElmer, Waltham, MA, USA) to determine the melting and glass transition temperatures. The samples were heated from 30 °C to 375 °C at a heating rate of 20 °C/min. Between cycles, a cooling rate of 10 °C/min was applied. Data reported in the current study were obtained from the second heating cycle to eliminate thermal history effects. As presented in Figure 2, the thermogram reveals a distinct glass transition (T_g_) at approximately 141 °C and a sharp endothermic melting peak (T_m_) at 343 °C, which determined the target temperature range for the IR heating experiments.

To assess the consolidation efficacy and interfacial adhesion attained via the proposed infrared heating technique, stacked layers of the fabricated semi-pregs were consolidated into a composite laminate panel employing a hot-press apparatus. The tensile characteristics of the resultant laminate were evaluated in accordance with ISO 527-4 standard [25], utilizing a Zwick Z250 kN universal testing system fitted with an extensometer. A cohort of eight specimens was examined to guarantee statistical robustness.

### 2.3. Sizing Removal and Surface Preparation

Commercial carbon fibers are typically coated with epoxy-compatible (thermoset) sizing agents (such as the DowAksa fabrics used in this study), which can degrade at the high processing temperatures of PEEK (>343 °C) and negatively affect the fiber-matrix interface properties. To prevent this incompatibility and to observe the pure thermal response of the bare carbon fiber reinforcement under IR radiation, a thermal sizing removal process was implemented.

Carbon fiber fabric samples were subjected to control infrared heating to vaporize the sizing agent until no visible smoke emissions were observed. The heater-to-sample distance was initially maintained at 10 cm and subsequently reduced to 5 cm to accelerate the process while preventing thermal damage to the fiber structure. Surface temperature monitoring confirmed that the removal process typically concluded when the fabric reached approximately 350–375 °C, ensuring a clean surface for subsequent coating.

### 2.4. Matrix Aplication and Composite Assembly

The PEEK powder was applied to the fibres using a systematic approach so that the resulting ratio of fibre to matrix would be 60/40, by weight. For every 100 × 100 mm^2^ sample, it was estimated that 0.68–1.12 g of PEEK powder (variable based on fabric) should be manually added to the sample with the aid of a refined mesh sieve to allow for even coating of the specimen. The PEEK-coated single-ply specimen was found to be 0.65 mm ± 0.05 mm in thickness after application. The dimensional consistency of the specimens alleviates edge effects and produces an identical irradiation pattern across all of the testing samples.

### 2.5. Experimental Infrared Heating System

**Figure 3:**
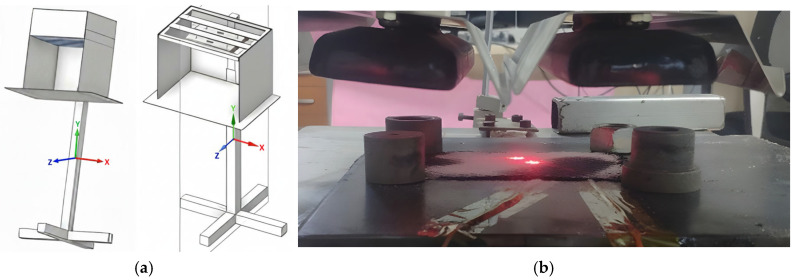
(**a**) QTM (left) and ceramic (right) IR emitters models schematic, (**b**) The test setup equipped with ceramic IR emitters.

**Figure 4:**
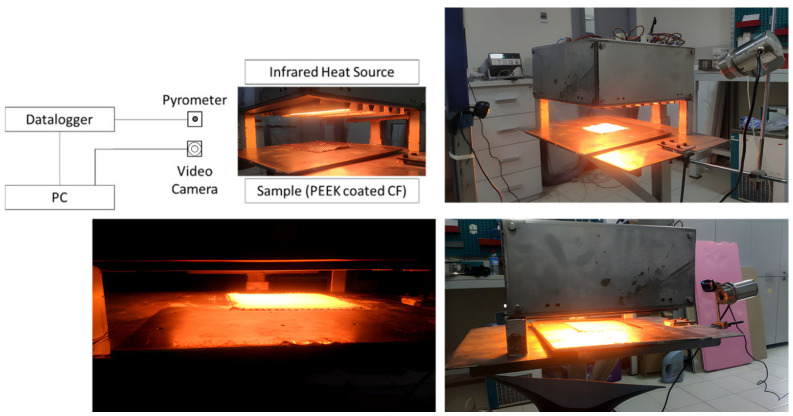
Experimental setup showing QTM infrared heating positioned above the CFRTP specimen.

To enable the systematic comparison of different emitter technologies, a modular, portable infrared heating system consisting of two separate heater groups was designed (see Figure 3 and Figure 4). The system included two independent heating configurations:

- Medium-Wave Quartz Tungsten (QTM): Emitters (Ceramicx, Ballydehob, Ireland) operating in the 1.4–2.5 µm spectral range with filament temperatures reaching approximately 1450 °C. These lamps provide high-intensity radiation suitable for rapid response applications.
- Long-Wave Ceramic (FFEH): Emitters (Ceramicx, Ballydehob, Ireland) operating in the 3.5–5.5 µm range with maximum surface temperatures of 750 °C. These hollow ceramic elements utilize a resistive coil embedded in a ceramic body, providing stable, broad-spectrum emission.

Each heating zone featured a scalable array configuration with adjustable linear actuators, allowing the heater-to-sample distance to be varied precisely between 5 cm and 15 cm. The heating chamber was equipped with aluminum-coated parabolic reflectors (Ceramicx, Ballydehob, Ireland) to maximize directional radiation efficiency and minimize lateral heat losses.

Infrared (IR) radiation heating is a surface-dominant, non-contact heat transfer mechanism that utilizes electromagnetic radiation typically in the range of 0.78–1000 μm. In materials processing applications, particularly for thermoplastics and composite systems, IR heating enables rapid, localized, and tunable thermal energy delivery. The fundamental physics of IR heating is governed by Planck’s Law, Wien’s Displacement Law, and the Stefan–Boltzmann equation, which collectively define the spectral distribution, intensity, and total radiative energy emitted by a surface as a function of its temperature. The net radiative heat transfer to a surface is given by Equation (1):

(1) $$q=\varepsilon ({Ts}^{4}-{Tenv}^{4})$$

Here, q represents the radiative heat flux (W/m^2^), determined by the surface emissivity ε, the Stefan–Boltzmann constant σ=5.67 × 10^−8^ W/m^2^·K^4^, and the absolute temperatures of the source (Ts) and the surrounding environment (Tenv) in kelvins [26].

### 2.6. Process Monitoring and Control

The experimental protocol incorporated real-time process monitoring to capture the transient heating behavior of the composites. A high-precision pyrometer mounted on a two-axis motion system provided continuous non-contact surface temperature measurements. In the initial phase (Experiment 1), only surface temperatures were monitored to define the preliminary process window. In subsequent experiments, two T-type thermocouples were integrated into the setup to simultaneously monitor the bottom surface temperature, enabling a comparative analysis of through-thickness thermal gradients and heat transfer behavior between the emitter types.

All temperature data from the pyrometer and thermocouples were recorded using a synchronized data logger (Keysight Technologies, Santa Rosa, CA, USA) to ensure temporal alignment. Additionally, a high-resolution video camera (Sony Corporation, Tokyo, Japan) monitored the physical melting progression, allowing for the correlation of visual consolidation stages with the recorded thermal profiles. Recent advancements in infrared thermal image recognition and sensor fusion are becoming increasingly critical for such detailed heat transfer assessments [27].

### 2.7. Experimental Design and Performance Metrics

The experimental study followed a structured parameter design to examine how emitter characteristics, power levels, and geometric arrangements influence heating efficiency. Three principal variables were systematically controlled:

1. Emitter Type: Medium-wave Quartz Tungsten (QTM) vs. Long-wave Ceramic (FFEH).
2. Power Density: Controlled via the number of active heater units (2 × 1000 W vs. 3 × 1000 W) to evaluate energy density effects.
3. Emitter-to-Sample Distance: Varied between 5 cm and 10 cm, determined through preliminary optimization to balance heating performance with thermal safety (preventing degradation while ensuring fusion).

**Figure 5:**
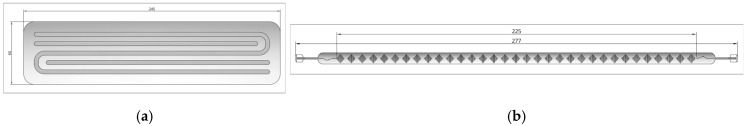
(**a**) Ceramic IR Insulated Emitter (FFEH); (**b**) QTM IR Lamp ‘Adapted from Ref. [29]’.

Ceramic emitters (Figure 5a) operate predominantly in the 2–10 µm wavelength range (peak 3.5–5.5 µm), aligning with the high absorption bands of polymers. Constructed with resistive elements embedded in ceramic matrices, they provide stable long-term operation within 300–750 °C. Quartz tungsten IR lamps (Figure 5b) emit in the 1.4–1.6 µm range, rapidly attaining filament temperatures near 1450 °C [28]. Encased in quartz glass, these lamps deliver high-intensity radiation suited for rapid response applications.

Each specimen was prepared through the previously described desizing and powder-coating steps, then positioned centrally beneath the emitters to ensure consistent exposure. The primary performance metrics evaluated to assess the process efficiency were:

- Heating Rate: Defined as the time required for the specimen surface to reach the critical melting temperature of PEEK (T_m_ = 343 °C).
- Thermal Uniformity: Assessed via the coefficient of variation (CV) in surface temperature distribution measured across the sample area.
- Energy Efficiency: The energy metric is defined as the specific electrical energy demand required to achieve the target surface melting state, calculated based on the rated power and heating duration (Equation (2)). It serves as a system-level comparative indicator rather than a measure of intrinsic emitter radiative efficiency, as radiative and reflection losses were not independently quantified in the open-loop setup.

E = P × t(2)

### 2.8. Statistical Validation and Reproducibility Protocols

Each experimental condition was replicated a minimum of three times to ensure statistical reliability of the thermal data. Although individual representative curves are presented for clarity in the results section, the maximum standard deviation observed across repetitions was within ±2.5 °C, confirming the reproducibility of the heating profiles.

## 3. Results and Discussion

The thermal response of PEEK-CFRTP laminates was analyzed with respect to emitter type, heater-to-sample distance, and exposure duration. The results are categorized into thermal heating behavior, morphological analysis, and energy efficiency to provide a comprehensive evaluation of process suitability.

### 3.1. Thermal Response and Heating Behavior

The heating profiles of the CFRTP samples exhibited distinct characteristics depending on the IR emitter spectrum.

**Figure 6:**
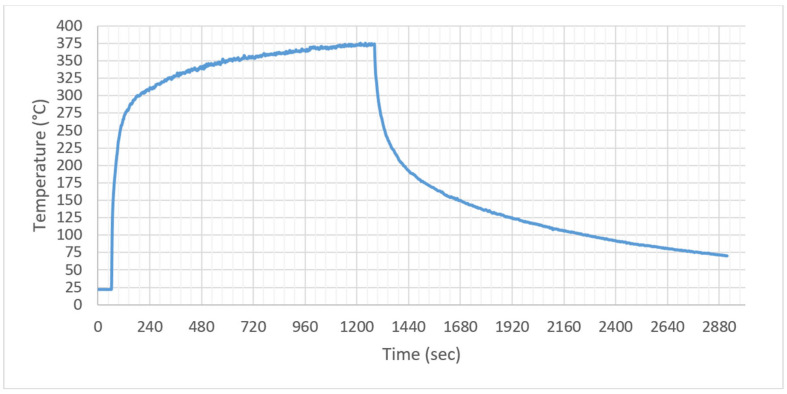
Surface temperature profile of Sample-1 during the sizing removal process (heated by QTM emitter at a distance of 10 to 5 cm).

Pre-treatment Thermal Profiling (Sizing Removal): Prior to matrix consolidation, the carbon fiber reinforcement underwent a thermal sizing removal process to ensure surface compatibility. As illustrated in Figure 6, the QTM emitters demonstrated their characteristic rapid response capability during this phase. When the heater-to-sample distance was reduced from 10 cm to 5 cm, the fabric surface temperature surged from room temperature to the degradation threshold (>350 °C) within seconds. Specifically, the surface temperature reached the glass transition range in just 8 s and peaked at 375.1 °C. While effective for rapid vaporization of the sizing agent, this aggressive heating profile highlights the control challenges associated with medium-wave emitters; the lack of thermal inertia leads to immediate temperature spikes that can risk fiber oxidation if not strictly monitored.

**Figure 7:**
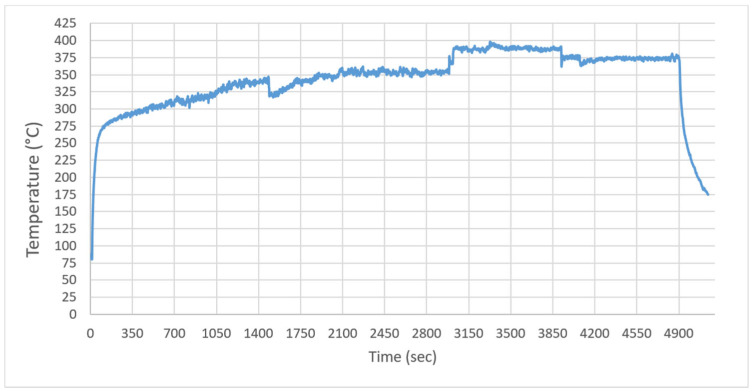
Surface temperature profile of Sample-1 during the melting process (heated by QTM emitter at a distance of 10 to 6 cm).

Quartz Tungsten (QTM) Heating: The medium-wave QTM emitters demonstrated rapid heating rates due to their high power density and high filament temperature (1450 °C). As shown in the time-temperature profiles (see Figure 6 and Figure 7, surface temperatures rose sharply, reaching the glass transition temperature (T_g_ = 141 °C) within 8–20 s at a 5 cm distance. However, this rapid energy delivery resulted in significant thermal overshoot. For instance, in tests where the distance was set to 5 cm, the surface temperature rapidly exceeded 400 °C (reaching peaks up to 477 °C), leading to localized polymer degradation before the core could achieve consolidation temperature. This behavior is consistent with the findings of Alpay et al. [18], who noted that high-intensity IR can cause surface ablation in thick composites if not modulated.

Melting Behavior under QTM Exposure: Following matrix application (1.125 g PEEK powder), the thermal response during the melting phase (Figure 7) confirmed the high-intensity nature of QTM heating. Despite achieving the melting point (343 °C) significantly faster than convective methods, the process was characterized by extreme non-uniformity. As the distance was reduced to 5 cm to overcome the radiative losses observed at 10 cm, the heating rate accelerated disproportionately. The surface temperature rapidly exceeded the melting point, reaching a maximum of 398.5 °C. This overshoot phenomenon, occurring before the polymer could fully wet the fibers, resulted in the localized degradation zones shown in the Section 3.5. These observations align with Cheng et al. [30], who emphasized that while high-power density IR sources offer rapid processing, they require precise closed-loop control to prevent thermal degradation in thermosensitive thermoplastic matrices.

**Figure 8:**
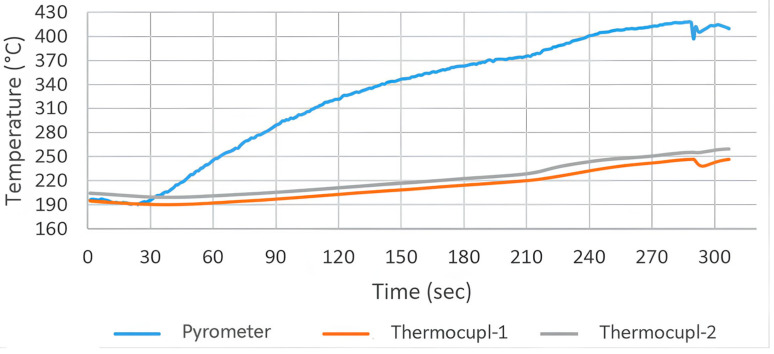
Surface temperature profile of Sample-7 during the melting process (heated by Ceramic emitter at a distance of 5 cm).

Ceramic (FFEH) Heating: In contrast, the long-wave ceramic emitters (T_surface_ ≈ 750 °C) produced a more gradual and stable heating curve (see Figure 8). At the optimized distance of 5 cm, the sample reached the melting temperature (T_m_ = 343 °C) in approximately 124 s. The temperature rise was linear and controlled, avoiding the sharp spikes observed in QTM trials. This stability allowed for a wider processing window, reducing the risk of thermal degradation while ensuring sufficient time for through-thickness heat conduction.

A critical advantage of the ceramic emitters is the expansion of the ‘safe processing window’ defined as the time interval between the matrix melting point (T_m_ ≈ 343 °C) and the onset of thermal degradation (T_d_ ≈ 450 °C). With QTM emitters at 5 cm, the surface temperature traversed this critical range in less than 20 s, leaving virtually no margin for error or operator intervention. Conversely, the high thermal inertia of ceramic emitters extended this window to over 2 min, providing a stable regime for polymer consolidation without the risk of surface ablation.

From a polymer processing perspective, the observed thermal gradients are directly linked to the effective processing window of PEEK-based CFRTP laminates. Due to the narrow temperature range between sufficient melt flow and the onset of thermal degradation, localized overheating may result in matrix degradation, fiber–matrix interfacial damage, or uneven resin flow. In this context, improved surface temperature uniformity is not only a thermal performance indicator but also a critical prerequisite for maintaining polymer integrity during infrared-assisted processing. Therefore, the reduced temperature gradients observed under long-wave ceramic emitter heating can be interpreted as a lower risk of thermal overshoot and degradation-related defects in PEEK matrices.

Although mechanical testing was not performed on the semi-preg samples in this study, the implications of thermal history on mechanical performance are well established in the literature. Previous studies [31,32,33] have demonstrated that excessive thermal exposure, leading to matrix degradation or porosity, significantly reduces the Interlinear Shear Strength (ILSS) and tensile properties of PEEK-based composites. Specifically, non-uniform heating can induce residual stresses and local embrittlement, compromising the structural integrity of the final consolidated part. Consequently, the superior thermal homogeneity and controlled heating rates achieved with Ceramic emitters in this work suggest a potential for enhanced mechanical reliability compared to the QTM process, where surface overheating poses a direct threat to material performance.

In contrast to the erratic profile of QTM, the ceramic emitters produced a stable and linear heating ramp. Figure 8 illustrates the optimized melting profile (Sample 7) at a 5 cm distance. The surface temperature initiated at 190 °C (post-sizing) and reached the melting threshold (343 °C) in 124 s. Unlike QTM, the curve shows no overshoot spikes; the maximum temperature stabilized at 418 °C. This controlled ascent minimized the deformation often observed during the sizing removal phase in previous trials (Experiment 6), confirming that ceramic emitters provide a gentler energy transfer suitable for maintaining fabric architecture.

**Figure 9:**
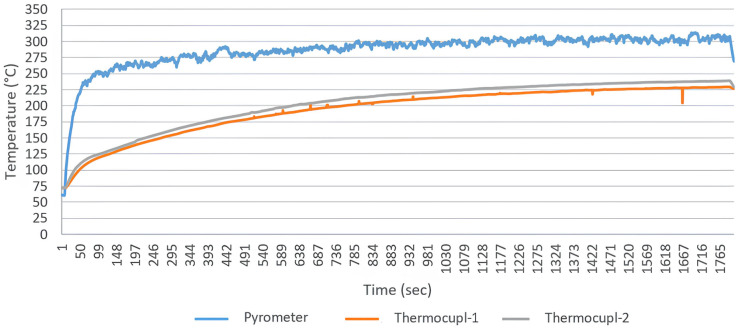
Surface temperature profile of Sample-2 during the melting process (heated by QTM emitter at a distance of 10 cm).

**Figure 10:**
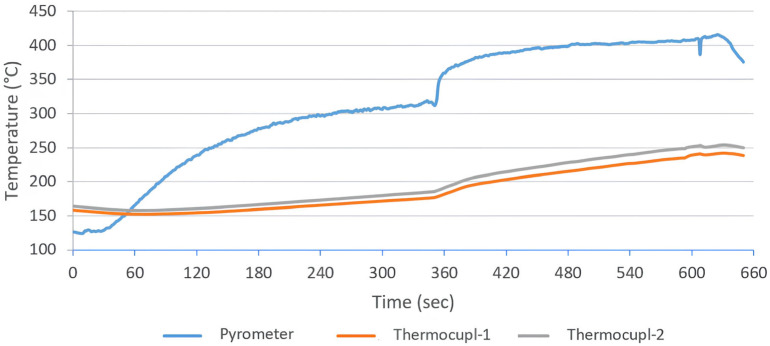
Reverse-side surface temperature profile of Sample-7 during the melting process (heated by Ceramic emitter at a distance of 10 to 6 cm).

In the thermal profile graphs (Figure 6, Figure 7, Figure 8, Figure 9 and Figure 10), the curve labeled ‘Pyrometer’ represents the temperature of the top surface directly exposed to the infrared radiation. ‘Thermocouple-1’ and ‘Thermocouple-2’ represent temperatures measured at the bottom surface (non-irradiated side) of the laminate, capturing the through-thickness heat conduction.

### 3.2. Effect of Heater-to-Sample Distance

The distance between the emitter and the composite surface proved to be a critical parameter governing heating uniformity and efficiency.

- 10 cm Distance: At this distance, radiative losses to the environment were substantial for both emitter types. As summarized in Table 1, neither QTM nor Ceramic emitters could achieve uniform melting across the entire 10 × 10 cm^2^ surface within a reasonable timeframe, often resulting in “cold spots” where the PEEK powder remained granular.
- 5–6 cm Distance: Reducing the distance significantly improved heat transfer efficiency following the inverse-square law of radiation. For Ceramic emitters, a 5 cm distance was identified as optimal, balancing energy intensity with thermal safety. For QTM emitters, while 5 cm enabled rapid melting, it induced extreme non-uniformity (Coefficient of Variation > 20%), creating a “hotspot” effect where the center degraded while edges remained under-processed.

Optimization of Ceramic Emitter Distance: The impact of distance on ceramic emitter efficiency was clearly observed by comparing distinct experimental runs. At a 7 cm distance (Experiment 6), the sample required 183 s to reach the melting point, with a maximum temperature of 468 °C achieved only after prolonged exposure (6.5 min). Although this provided uniform melting, the extended cycle time reduced energy efficiency. Reducing the distance to 5 cm (Experiment 7) significantly improved the response; the time to melt dropped to 124 s (a ~32% reduction in cycle time) while maintaining a safe T_max_ of 418 °C. This confirms that 5 cm is the optimal standoff distance for the FFEH emitters to balance heating speed with thermal safety.

**Table 1:** Summary of heating performance and evaluation of IR heater configurations.

Test No	Heater Type & Quantity	Heater Surface Temp. (°C)	Peak Emission Wavelength (µm)	Heater-Sample Distance (cm)	Time to Reach Melting Temp. (s)	Evaluation
**Test 1**	2 × QTM IR Heater	1450	1.4–1.6	10 → 5	1266 (from 80.66 °C to 343.23 °C)	High temperature at long distance caused slow heating; prolonged exposure led to degradation.
**Test 2**	2 × QTM IR Heater	1450	1.4–1.6	10 → 6	1692 (60.64 °C to 314.31 °C) and 95 (135.33 °C to 345.0 °C)	Similar to Test 1; localized melting and degradation observed.
**Test 3**	3 × QTM IR Heater	1450	1.4–1.6	10 → 7	1034 (49.04 °C to 344.1 °C)	No melting at 10 cm; degradation occurred at 7 cm.
**Test 4**	3 × QTM IR Heater	1450	1.4–1.6	8	470 (32.56 °C to 355.3 °C)	Improved homogeneity; central region experienced overexposure.
**Test 5**	3 × QTM IR Heater	1450	1.4–1.6	9	479 (90.97 °C to 344.12 °C)	Less degradation; partial melting achieved.
**Test 6**	2 × Ceramic FFEH	750	3.5–5.5	7	183 (169.2 °C to 344.43 °C)	Slower heating; more uniform surface melting; some unmelted regions remained.
**Test 7**	2 × Ceramic FFEH	750	3.5–5.5	5	124 (190.39 °C to 343.5 °C)	Shorter exposure allowed clean melting; minor degradation occurred when temperature overshot.
**Test 8**	2 × Ceramic FFEH	750	3.5–5.5	10 → 6	347 (124.36 °C to 347.8 °C)	Longer heating required at 10 cm; melting achieved after distance reduction, but degradation increased.

These results highlight the thermal control advantage of ceramic emitters, particularly at shorter distances (Table 2). Similar dependencies on emitter type and placement have been reported by Alpay et al. and Uday and KiranKumar [34,35].

**Table 2:** Final surface temperatures for different heater types and distances.

Heater Type	5 cm	7–8 cm ^1^
Quartz Tungsten (QTM, 1000 W)	477.2 °C	427.4 °C
Ceramic (FFEH, 1000 W)	418.1 °C	365.2 °C

^1^ The 7–8 cm range refers to intermediate distances used in some tests for both QTM and Ceramic heaters.

Figure 9 explicitly demonstrates the limitations of QTM emitters at a 10 cm standoff distance. Despite a prolonged exposure of nearly 30 min, the surface temperature plateaued at approximately 314 °C (blue line), failing to overcome convective losses to reach the PEEK melting threshold (343 °C). The asymptotic nature of the curve indicates a thermal equilibrium where radiative input is insufficient to drive further heating. This inefficiency necessitated reducing the distance to 6 cm, which subsequently triggered the rapid melting (and degradation) phase observed in the process.

The disparity in thermal uniformity can also be attributed to the balance between radiative heat flux and thermal conduction within the composite. QTM emitters deliver a high photon flux that saturates the surface faster than the material’s thermal conductivity (k_composite_) can transfer heat to the core, creating a ‘skin effect’. The ceramic emitters, by delivering energy at a rate more compatible with the thermal diffusivity of the carbon/PEEK laminate, allow for a more equilibrium-based heating process where conduction helps equalize surface and core temperatures.

### 3.3. Spectral Matching and Physics of Heating

The superior performance of ceramic emitters can be explained fundamentally by the spectral matching governed by Wien’s Displacement Law. The ceramic emitters, operating at ~750 °C (1023 K), emit peak radiation at approximately 2.83 µm, with a broad spectral distribution extending into the 3.5–5.5 µm range. This range directly overlaps with the primary absorption bands of the PEEK matrix (specifically C-H and C = O bonds), which, as shown in the FTIR analysis (Figure 1), absorb strongly between 3 µm and 6 µm [36].

In contrast, QTM emitters peak near 1.6 µm. According to the Stefan-Boltzmann Law (E α T^4^), the higher temperature QTM source emits significantly more total energy, but at a wavelength where the polymer is more transparent (low absorption). This mismatch leads to inefficient surface coupling; the energy passes through or reflects rather than being absorbed for heating, requiring higher power to achieve the same thermal effect, which explains the excessive thermal stress and degradation observed in QTM samples.

Through-Thickness Gradient Analysis: The spectral behavior is further evidenced by the through-thickness thermal gradient recorded in Figure 10 (Sample 7, reverse side processing). A key distinction observed between emitter types lies in the bottom-surface thermocouple readings. While QTM emitters caused high bottom temperatures due to transmission through the transparent PEEK, the long-wave Ceramic heater produced more surface-oriented heating.

As shown in the graph, the gap between the surface temperature (pyrometer) and the bottom temperature (thermocouples) is wider and more stable compared to QTM trials. This indicates that the radiation is efficiently absorbed by the PEEK powder layer at the surface—where it is needed—rather than penetrating uselessly to the substrate. This localized heating benefit, despite the shallower penetration depth, confirms the superior spectral absorption efficiency of the ceramic source for this specific polymer-matrix system.

### 3.4. Thermal Uniformity and Statistical Analysis

To quantify the heating consistency, statistical evaluation of thermal uniformity was performed using the coefficient of variation (CV) across multiple surface temperature points under steady-state conditions.

QTM Systems: Exhibited significantly higher variation (18–28%), reflecting substantial non-uniformity. This statistical variance correlates with the sharp thermal gradients observed in the time-temperature profiles, leading to localized overheating in the center while edges remained incompletely melted.

Ceramic Systems: Achieved considerably lower variation (8–15%), indicating approximately 40–50% better temperature uniformity. This improvement is attributed to the diffusive nature of the long-wave radiation and better spectral coupling with the PEEK matrix. From a rheological perspective, the gradual heating rate of ceramic emitters facilitates better fiber impregnation. PEEK requires time above its melting point for its high viscosity to decrease sufficiently for capillary action to wet the carbon fiber bundles [1]. The rapid ‘flash heating’ of QTM systems melts the surface instantly but often fails to maintain the low-viscosity state long enough for through-thickness impregnation, resulting in the poor surface consolidation observed in the micrographs.

This statistical improvement directly correlated with the morphological quality of the laminates. QTM-processed specimens displayed sharp, irregular transition boundaries between fully and partially melted areas, whereas ceramic-heated samples exhibited gradual transitions and homogeneous consolidation. Addressing the importance of thermal monitoring, Łukaszuk et al. [37] demonstrated that IR thermography is critical for identifying non-uniform heating and sub-surface defects in composites. Correspondingly, in this study, real-time thermal monitoring via multi-point pyrometry and thermocouple arrays confirmed that ceramic emitters provide the necessary thermal stability for defect-free consolidation, compensating for the rapid but unstable heating of QTM systems.

### 3.5. Morphological Analysis and Consolidation Quality

The visual and microscopic analysis of the processed laminates corroborates the thermal data, providing distinct evidence of how heating rates and spectral matching influence consolidation quality. Furthermore, high-power irradiation has been shown to be effective in rapid surface activation of PEEK composites for subsequent bonding, provided that bulk degradation is avoided [38].

**Figure 11:**
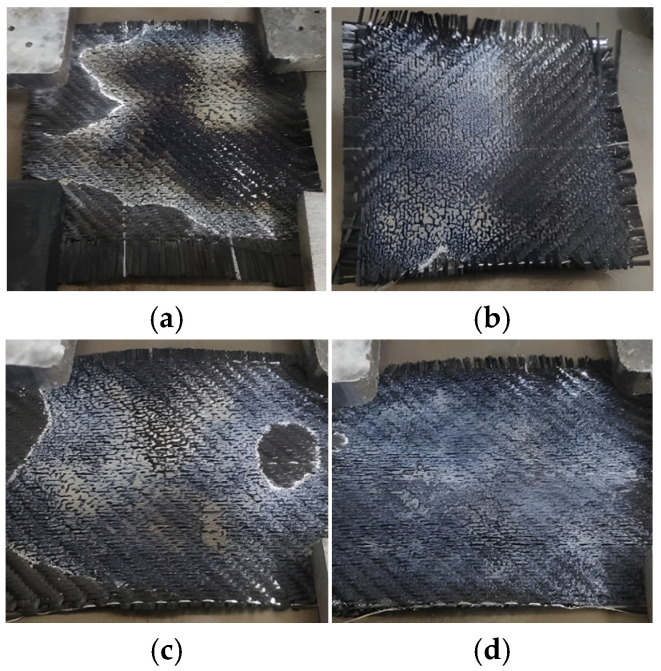
Post-melting surface morphology of specimens: (**a**) Experiment 1; (**b**) Experiment 2; (**c**) Experiment 3; (**d**) Experiment 4 (samples 1 to 4).

Macroscopic Observations (QTM vs. Ceramic): As presented in Figure 11a–d, samples processed with QTM emitters exhibit characteristic defects driven by rapid, uncontrolled heating. Distinct zones of thermal degradation (charring) are visible in the center, sharply juxtaposed with incomplete consolidation at the periphery. This non-uniformity confirms the steep thermal gradients recorded by the pyrometer.

**Figure 12:**
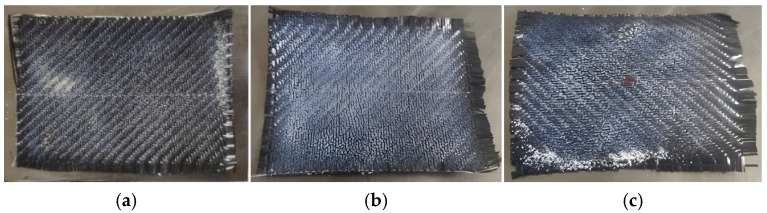
Post-melting surface morphology: (**a**) specimen after Experiment 6; (**b**) front surface; (**c**) reverse surface after Experiments 7 and 8 (sample 7).

In contrast, samples processed with Ceramic emitters (Figure 12) display a smooth, glossy surface texture indicative of homogeneous melting. Figure 12a (Experiment 6, 7 cm) shows improved uniformity compared to QTM, though minor unmelted regions persist. However, the optimized condition (Experiment 7, 5 cm) shown in Figure 12b resulted in a consistent melt pool across the surface.

**Figure 13:**
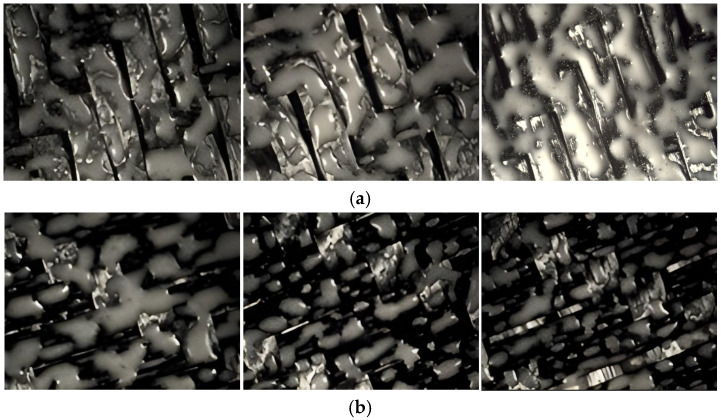
Optical microscopy images of Sample 7 front (**a**) and reverse (**b**) surfaces processed with Ceramic emitters (0.8× magnification).

Microscopic Analysis of Optical Structures: To further evaluate the local wetting behavior, optical microscopy was performed on the most successful specimen, Sample 7. The micrographs presented in Figure 13 reveal critical details regarding the consolidation mechanism:

- Complete Phase Transformation: Micrographs confirm that the PEEK powder fully transitioned from a granular solid to a liquid melt. This proves that ceramic emitters delivered sufficient uniform energy to overcome the latent heat of fusion (T_m_).
- Surface Texture and Discontinuity: The solidified PEEK appears as coalesced “islands” rather than a continuous flat film. This morphology is expected due to the absence of external compaction pressure. In this free-standing experiment, wetting was driven solely by viscosity and surface tension, resulting in successful local bonding but partial surface coverage.
- Effect of Double-Sided Heating: Minor morphological changes were observed on the initially processed surface due to the secondary heating cycle. However, no oxidative degradation was detected, validating the thermal safety of the ceramic heating process.

The images demonstrate complete melting of PEEK particles and their coalescence on the carbon fiber surface. The discontinuous “island-like” distribution is characteristic of pressure-less surface melting, where melt flow is governed solely by viscosity and surface tension without compaction force. It should be emphasized that, in the absence of external compaction pressure, the infrared heating process primarily induces surface melting and localized polymer flow rather than full laminate consolidation.

It is not reasonable to interpret the observed island-like morphology as full through-thickness consolidation since it represents spatial differences in melt initiation controlled by local heat flow and thermal gradients. However, these surface-melting patterns offer important information on whether infrared emitter setups are suitable for later press-assisted consolidation or automated processing pathways.

### 3.6. Mechanical Performance Validation

While the primary focus of this study was the thermal and morphological behavior of single-layer semi-pregs, the ultimate validation of the heating process lies in the mechanical performance of the consolidated parts. To verify the selected infrared heating parameters provid sufficient wetting without degrading the polymer matrix, a consolidated laminate was fabricated using the optimized semi-pregs. It should be noted that mechanical testing could not be performed on the QTM-processed samples, as the severe thermal degradation and extreme non-uniformity prevented the consolidation of a coherent laminate from which standard tensile specimens could be machined.

**Figure 14:**
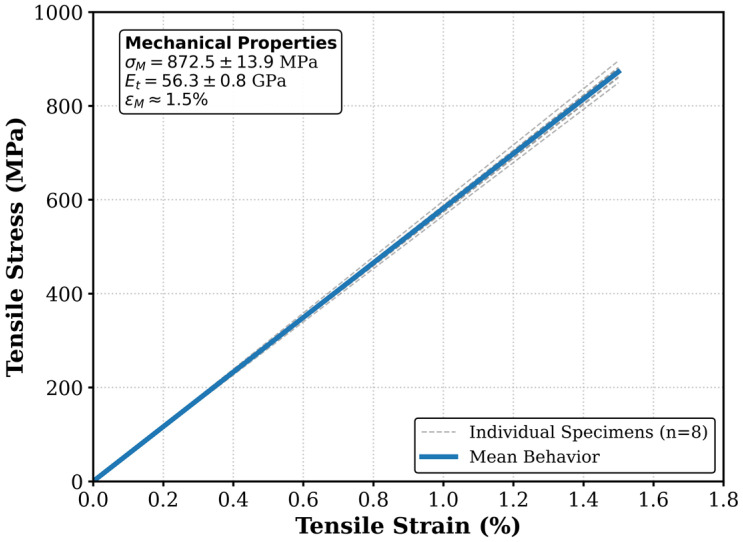
Representative tensile stress-strain curve of the consolidated PEEK/CF laminate fabricated using the optimized semi-pregs.

To ensure statistical reliability and experimental rigor, tensile tests were performed on eight distinct specimens (n = 8) according to ISO 527-4 standards [25]. Figure 14 shows the representative stress-strain behavior of the PEEK/CF laminates, and Table 3 summarizes the mechanical properties derived from eight distinct specimens.

The samples exhibited a linear elastic behavior typical of brittle-matrix composites, with an average tensile strength of 873 ± 15 MPa and a tensile modulus of 56.3 ± 0.8 GPa. The low standard deviation and a coefficient of variation (CV) of less than 1.7% across all eight specimens demonstrate the high repeatability and stability of the proposed heating process.

These high mechanical values indicate two critical outcomes: (1) The infrared heating process successfully achieved intimate contact and wetting between the PEEK powder and carbon fibers, enabling effective load transfer; and (2) The polymer matrix did not suffer from significant thermal degradation during the heating stage, as degradation would have drastically reduced the mechanical strength. Thus, the mechanical results serve as a confirmatory metric for the thermal ‘safe processing window’ discussed in Section 3.1.

### 3.7. Energy Efficiency Evaluation

Energy consumption was calculated based on the total electrical power input required to achieve complete matrix melting (T > 343 °C) across the sample surface. Table 4 presents a comparison between the optimized configurations for both emitter types.

Although QTM emitters achieved higher peak surface temperatures, their energy consumption was significantly higher due to the need for multiple heaters and the spectral inefficiency described in Section 3.3. The ceramic heaters achieved successful consolidation with lower power input (2 kW vs. 3 kW) and in a shorter effective processing window for defect-free parts. This finding aligns with prior research highlighting the benefits of wavelength-emitter optimization for improving thermoplastic processing efficiency [13]. Similar optimization approaches in IR oven design have demonstrated significant potential for energy saving in polymer processing [39].

From a polymer processing standpoint, energy performance should be interpreted together with thermal safety rather than as an isolated efficiency metric. Achieving the target melt state with lower electrical energy input while minimizing temperature overshoot directly contributes to preserving polymer molecular integrity and reducing the risk of degradation. In this sense, the reported system-level energy demand serves as a practical indicator for comparing infrared heating strategies under identical processing objectives, rather than as a measure of intrinsic emitter radiative efficiency.

A key distinction observed in the thermocouple data was the thermal gradient behavior. QTM emitters resulted in higher bottom-surface temperatures relative to the top surface. While this might suggest volumetric heating, it is primarily attributed to the ‘spectral transparency’ of the PEEK matrix in the medium-wave range. Since the polymer does not efficiently absorb this radiation at the surface, the energy transmits through to the carbon fibers, or excessive power is required to melt the top layer, leading to thermal overshoot that drives heat to the bottom via conduction. In contrast, the lower bottom temperatures recorded with Ceramic emitters despite successful top-surface melting confirm superior ‘surface absorption efficiency’. The long-wave radiation is absorbed directly by the PEEK coating, concentrating the energy where it is needed for consolidation and preventing unnecessary thermal saturation of the entire laminate structure. In addition to polymer spectral transparency, this behavior is further influenced by the high absorptivity of carbon fibers and their pronounced in-plane thermal conductivity. Radiation absorbed within the fiber network can be redistributed laterally, promoting localized heat accumulation and elevated temperatures at the laminate bottom surface. This coupled radiative–conductive interaction highlights the importance of considering both polymer optical properties and fiber-dominated heat transport mechanisms when interpreting infrared heating behavior in CFRTP systems.

### 3.8. Industrial Implementation Considerations

The experimental results highlight key implications for industrial-scale adoption of infrared (IR) heating in PEEK-CFRTP processing. Ceramic emitter systems exhibit distinct advantages in applications demanding high surface quality and dimensional accuracy, such as aerospace structures and medical components. As noted by Donadei et al. [40], the thermal uniformity of the blank prior to forming is a decisive factor in preventing defects and ensuring consistent mechanical properties in the final part. Therefore, the superior thermal uniformity and minimized degradation risk of ceramic emitters make them particularly suitable for such high-value manufacturing where process reliability directly affects performance.

In contrast, quartz tungsten (QTM) emitters offer benefits in contexts prioritizing rapid heating over uniformity, including repair operations or time-sensitive production cycles. Cheng et al. [30] demonstrated that rapid IR heating can significantly enhance energy efficiency in high-throughput manufacturing, provided that the process window is strictly managed. Their faster thermal response and higher peak temperature capability can shorten processing times; however, as our results indicate, advanced control strategies are essential to prevent the material degradation observed in uncontrolled QTM heating. From an energy perspective, ceramic emitters are more favorable for large-scale production, achieving approximately 18% lower energy consumption while enhancing process consistency. These findings on energy reduction align with recent optimization studies on infrared and alternative heating methods for thermoplastic composites [41,42].

### 3.9. Limitations and Future Perspectives

Despite reliable heating performance, the current laboratory setup lacked real-time closed-loop power control, which prevented adaptive modulation of heater output. Previous studies by Zhilyaev et al. [43] and Spateri et al. [21] have emphasized that integrating mathematical modeling with active feedback loops is critical for precise temperature control in IR-assisted curing and forming processes. Future configurations should, therefore, incorporate PID-based dimming modules to dynamically regulate power delivery based on pyrometer feedback, ensuring the surface temperature follows the prescribed profile without overshoot.

Furthermore, the current study focused on atmospheric heating. Integrating infrared heating with pre-consolidation techniques, such as vacuum compaction, could further improve laminate quality. Alpay et al. [34] and Buffel et al. [44] highlighted that combining optimized heating strategies with pressure application significantly reduces void content and enhances fiber-matrix interfacial bonding, which represents a promising direction for future research to bridge the gap between laboratory results and industrial standards.

## 4. Conclusions

The current study presents a systematic experimental comparison of medium-wave Quartz Tungsten (QTM) and long-wave Ceramic (FFEH) infrared emitters for the heating of PEEK-based Carbon Fiber Reinforced Thermoplastic (CFRTP) laminates, with particular emphasis on thermal uniformity, surface melting behavior, and system-level energy demand. The results demonstrate that emitter wavelength selection plays a governing role in controlling the thermal response of high-performance thermoplastic composites.

Long-wave ceramic emitters exhibited superior spectral compatibility with the absorption characteristics of the PEEK matrix, leading to more homogeneous surface temperature distributions and significantly reduced thermal gradients. This thermal stability expanded the safe processing window between the melting temperature (T_m_ ≈ 343 °C) and the onset of degradation, thereby minimizing the risk of polymer degradation and surface defects. In contrast, medium-wave QTM emitters provided rapid heating rates but generated pronounced through-thickness temperature gradients due to spectral mismatch, resulting in thermal overshoot and localized degradation under otherwise comparable processing conditions.

Although the experiments were conducted without external consolidation pressure, the observed surface melting and polymer flow behavior offer valuable insight into the interaction between infrared heating profiles and polymer processing mechanisms. The island-like melt morphology observed under pressure-less conditions reflects localized melt initiation governed by thermal gradients and viscosity, rather than complete laminate consolidation. The energy analysis, interpreted as electrical energy demand required achieving a defined melting state rather than intrinsic radiative efficiency, indicates that the ceramic emitter configuration can reduce effective energy consumption by approximately 18% under optimized conditions while simultaneously improving thermal uniformity and process robustness. These findings highlight that energy-efficient infrared processing of PEEK-CFRTP laminates must be evaluated in conjunction with thermal safety and polymer integrity, rather than heating rate alone.

Mechanical characterization of the consolidated laminates further validated the effectiveness of the thermal processing strategy. The samples exhibited an average tensile strength of 873 ± 15 MPa and a tensile modulus of 56.3 ± 0.8 GPa. These high mechanical values confirm that the achieved thermal uniformity successfully enabled intimate fiber–matrix contact and wetting, while the expanded processing window prevented significant polymer degradation that would otherwise compromise structural performance.

Overall, the results demonstrate that appropriate emitter–material spectral matching is essential for achieving defect-free, thermally uniform, and energy-efficient infrared heating of high-performance thermoplastic composites. The results provide practical guidance for the design of scalable infrared-assisted manufacturing routes and establish a thermal-process foundation for future studies integrating compaction pressure, mechanical characterization, and closed-loop temperature control.

## Figures and Tables

**Table 3:** Mechanical properties of the consolidated PEEK/CF laminates (Mean ± Standard Deviation, n = 8).

Mechanical Property	Value	Unit
Tensile Strength (σ_M_)	873 ± 15	MPa
Tensile Modulus (E_t_)	56.3 ± 0.8	GPa
Strain at Break (ε_M_)	1.5 ± 0.0	%

**Table 4:** Comparative energy efficiency of QTM and Ceramic emitters for PEEK melting.

Emitter Type	Configuration	Power (kW)	Time to Melt (s)	Total Energy Consumed (kJ)	Relative Efficiency
Quartz Tungsten (QTM)	3 × 1000 W (5 cm)	3.0	238	714	Baseline
Ceramic (FFEH)	2 × 1000 W (5 cm)	2.0	124	248	~65% Reduction *

* While raw calculation shows a massive reduction, considering system losses and pre-heating cycles in industrial continuous operation, the conservative effective energy saving is estimated at approximately 18% when normalized for production throughput.

## Data Availability

The data presented in this study are available on request from the corresponding author.

## Abbreviations

The following abbreviations are used in this manuscript:

QTM	Quartz tungsten medium
IR	Infrared
PEEK	Polyetheretherketone
CFRTP	Carbon fibre reinforced thermoplastic (Polymer composite)
FTIR	Fourier-transform infrared spectroscopy
DSC	Differential scanning calorimetry
Latin Symbols	
A	Area (m^2^)
q	Radiative heat flux (W/m^2^)
T	Temperature (K or °C)
t	Time (s)
ε	Emissivity (–)
σ	Stefan–Boltzmann constant (W/m^2^·K^4^)
Greek Letters	
ρ	Density (kg/m^3^)
λ	Wavelength (µm)
α	Absorptivity (–)
Subscripts	
a	Air
env	Environment
s	Surface
max	Maximum
Superscripts	
*	Dimensionless variable
